# Differential effects on blood and cerebrospinal fluid immune protein markers and kynurenine pathway metabolites from aerobic physical exercise in healthy subjects

**DOI:** 10.1038/s41598-021-81306-4

**Published:** 2021-01-18

**Authors:** Josef Isung, Mathias Granqvist, Ada Trepci, Jesse Huang, Lilly Schwieler, Marie Kierkegaard, Sophie Erhardt, Jussi Jokinen, Fredrik Piehl

**Affiliations:** 1 Centre for Psychiatry Research, Department of Clinical Neuroscience, Karolinska Institutet, and Stockholm Health Care Services, Region Stockholm, Stockholm, Sweden; 2 Division of Physiotherapy, Division of Neurology, Department of Clinical Neuroscience, Karolinska Institutet, and Academic Specialist Center, Stockholm Health Care Services, Region Stockholm, Stockholm, Sweden; 3 Department of Physiology and Pharmacology, Karolinska Institutet, Stockholm, Sweden; 4 Department of Neurobiology, Care Sciences and Society, Karolinska Institutet and Academic Specialist Center, Stockholm Health Care Services, Region Stockholm, Stockholm, Sweden; 5 Department of Clinical Sciences, Umeå University, Umeå, Sweden

**Keywords:** Biomarkers, Molecular medicine, Immunology, Neuroimmunology, Medical research

## Abstract

Mounting evidence shows that physical exercise modulates systemic inflammation. However, its effect on cerebrospinal fluid (CSF) immune-marker profiles in man are largely unknown. We here report a study on healthy subjects (n = 27, males = 12, mean age 28.7, range 22–52) allocated to either an acute exercise setting over four consecutive days, or a training intervention over 4 weeks. Paired plasma and CSF samples collected at baseline, after 7 days of exercise abstention, and the day after completion of the exercise interventions, were analyzed for protein inflammation markers using a multiplex proximity extension assay and neurotransmitters and kynurenine pathway (KP) metabolites using liquid chromatography, respectively. Routine cell counts, and albumin, immunoglobulin G and neurofilament light chain concentrations in CSF remained unchanged in both paradigms, while several inflammatory proteins became upregulated after acute exercise. However, only changes in three CSF (vascular endothelial growth factor-A, interleukin-7 and matrix metalloproteinase-10) and 12 plasma proteins reached significance levels after adjustment for multiple comparisons and exclusion of less stable proteins. Similarly, KP metabolites only changed among participants after acute exercise, while neurotransmitter levels, except for increased CSF serine, remained stable. Both in plasma and CSF changes in KP metabolites and inflammatory proteins correlated, suggesting that these processes are functionally linked. These findings suggest that acute aerobic physical exercise affects immune markers and KP metabolites systemically and in the CSF.

## Introduction

Physical exercise confers benefits across a wide range of diseases, including psychiatric, neurological, metabolic, cardiovascular, pulmonary diseases, and cancers^1,2^. The underlying molecular mechanisms are multifactorial, and remains to be established in detail. Several models have been proposed, including the release of myokines such as interleukin (IL)-6 and fibroblast growth factor (FGF)-21 from contracting muscles, expression of mitochondrial-specific transcription factors such as peroxisome proliferator-activated receptor gamma coactivator 1-alpha1 (PGC1α), increased neurotrophic factor synthesis and monoamine and glutamate (GLU) regulation^3,4^. Exercise even positively affects the mental state in healthy individuals, thought to involve changes in neuronal activation patterns^5^.

Evidence suggests that the neuropsychiatric effects of physical exercise relates to modulation of inflammatory activity^6,7^, although an exact mechanistic understanding of the link between physical activity, modulation of inflammatory responses and clinical effects is currently lacking^8^. This is likely in part related to the complexity of processes connecting muscle activity with effects mediated within the central nervous system (CNS)^4^. However, an interesting mechanism linking muscle activity to regulation of inflammation in the brain, in turn affecting susceptibility to stress-induced depressive behavior in mice was described by Agudelo et al.^9^. In this study activity of the transcription factor PGC1α in skeletal muscle of mice was shown to regulate the kynurenine pathway (KP) systemically and in the brain, which could be mechanistically linked to reduced expression of pro-inflammatory cytokines in the hippocampus and higher resilience to stress-induced depressive behavior. The KP involves a number of downstream metabolites of tryptophan (TRP), several of which are neuroactive through agonism or antagonism at classical neurotransmitter receptors known to be associated with mood disorders, such as the *N*-methyl-d-aspartate (NMDA) receptor^10^. This also provides a possible molecular background to effects on neuronal activation patterns that has been described in association with physical exercise, sometimes referred to as flow^11^. Alterations of central levels of KP metabolites have been described in a number of psychiatric disorders^12–16^, and neurodegenerative diseases, such as Alzheimer’s disease, Parkinson’s disease, Huntington’s disease, amyotrophic lateral sclerosis^10^ and multiple sclerosis^17^. Existing evidence on the effects from exercise on KP metabolites in the peripheral compartment in humans, suggest that acute bouts of exercise, rather than exercise over a longer time-frame induces dynamic alterations on the KP^18^. These dynamic changes are believed to result in an increased clearance of peripheral kynurenine, preventing KP metabolite accumulation in the CNS, and thus acting neuroprotective. Even if the KP represents a promising candidate pathway for mediating neuropsychiatric effects of physical exercise, it is still unknown if KP metabolites in the human intrathecal compartment indeed are modulated by physical exercise. Thus, assessing the dynamic changes in blood as well as the cerebrospinal fluid (CSF) in healthy individuals as an effect from exercise, also assessing in relation to different exercise paradigms is highly motivated. Similarly, there is a paucity of studies addressing effects of physical exercise on CSF immune-marker profiles. In a study by Schön et al., which focused on the acute effects of intense aerobic exercise on the insulin-adiponectin system in healthy individuals, CSF levels of adiponectin was shown to be downregulated after exercise while changes in cytokine levels generally were small^19^. However, the fact that CSF sampling was separated by a 4-week interval and that only six subjects provided paired samples likely affected the power to detect relevant differences. In another study, using a non-paired design with a separate set of controls, increases in IL-6 and heat shock protein-72 in blood of the exercise group were found not to correspond to similar increases in the CSF, suggesting that the two compartments are regulated separately^20^.

In summary, to date, there is only very limited information on to what extent physical exercise in humans might induce changes to the CSF immune protein marker profile, and virtually no information on its effect on the KP. Biomarker studies usually compares clinical populations with healthy controls, which may differ in physical activity level and exercise habits. The lack of knowledge in how exercise may impact immune markers and KP metabolites in the CSF and to what degree this is reflected by corresponding changes in the peripheral compartment represents an uncertainty in biomarker studies; therefore, representing a potential systematic bias. The two main objectives of the study were to assess the dynamic variability of immune markers as a consequence of physical exercise both in blood and CSF to shed light on molecular pathways affected by exercise and to address the potential of differences in exercise habits as a bias in biomarker studies comparing clinical populations compared to healthy controls.

## Methods

### Study population

Healthy participants were recruited from student campuses by advertising. The following eligibility criteria were applied; age $\ge$ 18 years. Exclusion criteria were; any physical condition constituting a hindrance to engage in the study procedures (exercise and CSF sampling), past or current major psychiatric disorder, or significant concomitant diseases such as autoimmune, inflammatory or infectious conditions, known heredity in first-degree relatives for severe psychiatric disorders like schizophrenia, bipolar disorder, major depression and/or suicidal behavior.

### Ethics approval

All participants provided written informed consent before enrolment and the study protocol was approved by the Regional Ethical Review Board in Stockholm, Sweden (Dnr 2014/1201-31/1) and was conducted according to the Declaration of Helsinki.

### Study protocol

**Figure 1:**
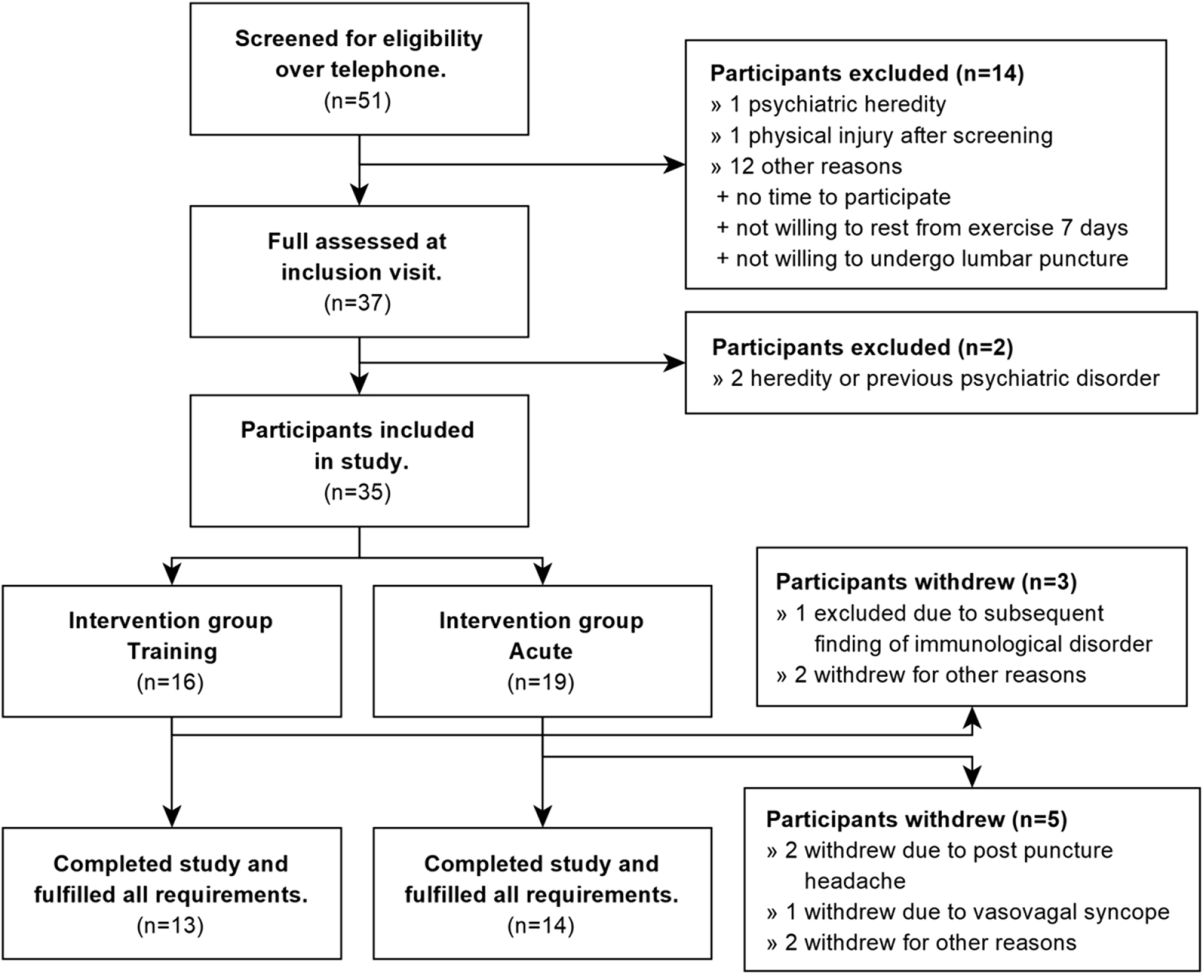
Study flowchart.

Interested participants were first screened by telephone and then scheduled for an inclusion assessment with a consultant psychiatrist where a full medical history was obtained. The diagnostic psychiatric MINI-International Neuropsychiatric Interview^21^ was performed to screen for common psychiatric disorders. Participants were further assessed on current health status, including a clinical basic examination. Thirty-five of 51 screened individuals were eligible for the study (Fig. 1). Based on their current level of habitual physical exercise, (weekly frequency, and intensity of exercise in the previous 6 months), study participants were allocated to one of two groups; four consecutive days of acute vigorous exercise or a training intervention requesting exercise of moderate-intensity three times weekly over 4 weeks. The Borg’s rating of perceived exertion (RPE scale), which ranges from 6 (nothing at all) to 20 (maximum), was used to guide exercise intensity^22^. Moderate intensity corresponds to RPE 12–13, vigorous to RPE 14–17, and near-maximal to maximal intensity corresponds to RPE ratings ≥ 18^23^. The Borg scale is a validated rating tool and has shown good correlation between perceived and physiological exertion variables^24,25^.

Participants who exercised ≥ 3 times/week and where exercise habits included long paced runs (up to 60 min) on at least vigorous intensity as well as high-intensity interval training or resistance training were allocated to the acute setting. This included running sessions 4 days in a row. On days one and three, participants were instructed to perform a 30-min high-intensity interval-training regime, i.e. 10 intervals of one-minute high intensity run (Borg RPE 18–19) followed by two minutes of low intensity run (Borg RPE (9–11). On days two and four, they were instructed to run at a vigorous pace for at least 60 min (RPE average 14–16). Participants who exercised ≤ 2 times/week and where exercise habits included physical activities at low-to-moderate intensity, such as power walking or slow jogging, were allocated to the training intervention. This included a minimum of 30 min running at a vigorous intensity (Borg RPE 14–16) three times weekly for 4 weeks, i.e. a total of 12 sessions. Participants were instructed to adhere to the exercise protocol and fill a standardized diary on exercise adherence that was provided before the post-exercise sampling.

Participants were instructed to abstain from any physical exercise 7 days before baseline sampling; the rationale for this being to limit any acute influence from recent exercise on biomarker levels. Baseline samples in blood and CSF was performed before initiation of the exercise intervention; whereas post-exercise samples were obtained the day after the last exercise session. Blood sampling was performed in the morning between 7.30 and 9 am while fasting since midnight and after a full night of bed rest. Blood was drawn and collected in a BD vacutainer plasma tube (9 ml) and allowed to clot for 30 min at room temperature before centrifugation (2900 rpm/1692rcf for 15 min). Lumbar punctures were performed according to standardized procedures using an atraumatic needle (22G, 70 mm) in the L4–5 or L5-S1 intervertebral space. A total volume of 25 ml of CSF was collected with separation of cells and freezing of supernatant at – 80 C within 1 h. All analyzes were run on samples that had not been previously thawed. In addition, routine CSF and blood analyses on fresh samples were performed at the Departments of Clinical Chemistry and Clinical Immunology of the Karolinska University Hospital and included cell numbers, albumin, immunoglobulin (Ig)G, isoelectric focusing and neurofilament light (NFL) chain protein concentrations. These Karolinska University Hospital laboratories are hospital-based services, and all analyses assayed are accredited according to ISO standard 15189. The NFL analyses were done with the NF-light ELISA according to instructions by the manufacturer (UmanDiagnostics, Umeå, Sweden).

### Multiplex proximity extension assay in CSF and plasma

The CSF and plasma proteins were determined using a Proseek Inflammation I^96×96^ proximity extension assay (PEA), which uses a proximity extension technology to enable a high throughput multiplex proteomic immunoassay (Olink, Uppsala, Sweden). Detailed descriptions of the assay methodology and preprocessing have been provided previously^26^. In short, the panel includes 92 cytokines and chemokines, and a selection of other immune-related proteins. The assay utilizes epitope-specific binding and hybridization of a set of paired antibodies linked to oligonucleotide probes, which subsequently can be amplified using a quantitative polymerase chain reaction to quantify relative protein concentrations in terms of log base-two normalized protein expression (NPX) values.

### LC/MS analyses of CSF kynurenine pathway metabolites and neurotransmitters

The CSF samples were analyzed for TRP, kynurenine (KYN), 3-hydroxykynurenine (3HK), picolinic acid (PIC), quinolinic acid (QUIN) using ultra performance liquid chromatography -tandem mass spectrometry system (LC/MS). For a detailed description of the analysis, see^27^. High performance liquid chromatography (HPLC) with fluorescence detection was used for analyses of kynurenic acid (KYNA), GLU, gamma-aminobutyric acid (GABA) and serine. To analyze KYNA a mobile phase of 50 nM sodium acetate (pH  6.2) and 7% acetonitrile was used. The flow rate was 0.5 ml/min and 20 μL of CSF sample was injected manually into the system. Signals from the detector were send to the computer and the software Datalys Azur (version 4.6.0.0) was used for analyses (Erhardt et al., 2000). To determine GLU, GABA and serine, a mobile phase (A) of sodium acetate buffer with a concentration of 0.04 M and ph = 6.95, with 2.5% methanol and 2.5% tetrahydrofuran; and another mobile phase (B) of methanol were used. Participants’ CSF samples were derivatized with *O*-phthaldialdehyde/2-mercaptoethanol reagent in room temperature for 60 s.

### Statistical analysis

Data were analyzed using multi-variable linear regression models adjusting for sex and age at sampling, as previously described^28^. In brief, differences in protein and KP metabolite levels pre-/post-intervention across the different exercise groups were analyzed using a paired Student *t* test. Potential effects of pre-analytical variability relating to possible disparity in sample handling was assessed for each measure and primary associations were analyzed using a multi-variable linear regression model adjusting for sex and age at sampling and along with curated markers of sample handling, chemokine (C–C motif) ligand 19 (CCL19) (CSF) and axin-1 (plasma)^29^. Associations were corrected for multiple testing using an FDR approach with a significance cutoff of P_FDR_ < 0.05. All statistical analyses and figures were computed in R-3.2.3 (Vienna, Austria). Statistical significance after correcting for multiple testing was considered for *p* values below 5 × 10^–5^.

## Results

### Study sample and routine CSF analyses

Twenty-seven of 35 eligible participants fulfilled the exercise programs, providing diary details regarding type, length and intensity of all exercise sessions, and contributed blood and CSF samples (Fig. 1). Participant characteristics for the total study sample (n = 27), and by exercise program, i.e. the 14 allocated to the acute exercise group and the 13 allocated to the training exercise group are presented in Table 1.

**Table 1:** Participant characteristics for the total study sample and by allocation to exercise program, i.e. the high-intensity and moderate-intensity exercise program.

	Total study sample (n = 27)	Acute group (n = 14)	Training group (n = 13)
Sex (n = male/female)	12/15	6/8	6/7
Age (years)^a^	28.7 ± 7.7, 22–52	28.1 ± 8.7, 22–52	29.3 ± 6.8, 22–45
Body Mass Index (kg/m^2^)^a^	22.6 ± 2.6, 18.9–29.6	22.9 ± 2.7, 19.6–29.6	22.2 ± 2.5, 18.9–28.0
Smokers	0	0	0
History of or active substance use disorder	0	0	0
Continous medication	0	0	0
Exercise habits^b^ (low, moderate, high)	Low (n = 6) Moderate (n = 7) High (n = 14)	High (n = 14)	Low (n = 6) Moderate (n = 7)

^a^Mean ± s.d., range.

^b^Self-reported measure of recent exercise activity.

Due to an error in blood sampling, three participants had no plasma stored; therefore, the plasma group consists of 24 participants, 12 in each exercise group respectively. All KP metabolites were successfully detected in all participants but one. In the analysis of both baseline and post-exercise CSF from this individual, the coefficient of variation in the detection of duplicates for PIC was more than 10% and, therefore, the samples from this individual were excluded from the statistical analyses. As for GABA, this was not detectable in baseline samples from two participants, as well as two additional participants at follow up, all four allocated to the training exercise group.

“Routine CSF analyses” revealed none or only modest changes before and after exercise (Table 2). The changes seen were more pronounced in the acute exercise group, with a non-significant drop in CSF IgG and albumin concentrations of 11% and 14%, respectively. The NFL concentrations were stable in the acute group, but displayed a modest, non-significant increase with 7% in the training group. No participant displayed CSF selective oligoclonal bands.

**Table 2:** Cerebrospinal fluid (CSF) routine markers and neurofilament light (NFL) before and after intervention.

	Sampling time	Training group^a^ (n = 13)	Acute group (n = 14)
Days between paired sampling,blood sampling and lumbar puncture^b^	–	49.1 ± 22.3, 28–97	10.6 ± 10.3, 4–41
CSF albumin/serum albumin quotient, reference < 7^c^	Baseline	4.6 ± 0.5, 2.7–7.9	5.1 ± 0.6, 2.2–9.5
	Follow-up	4.5 ± 0.5, 2.1–7.3	4.5 ± 0.5, 1.7–7.5
IgG index^c^	Baseline	0.48 ± 0.01, 0.42–0.54	0.48 ± 0.01, 0.37–0.52
	Follow-up	0.50 ± 0.006, 0.45–0.54	0.50 ± 0.007, 0.46–0.55
Serum albumin^c^	Baseline	45.5 ± 1.3, 39–52	43.8 ± 0.9, 37–50
	Follow-up	44.2 ± 0.9, 37–48	43.9 ± 1.2, 37–50
CSF albumin^c^	Baseline	206.8 ± 22.1, 116–364	228.9 ± 29.5, 91–432
	Follow-up	200.8 ± 22.2, 89–349	197.4 ± 24.1, 74–351
CSF IgG^c^	Baseline	23.8 ± 2.4, 16–43	26.2 ± 3.8, 8–62
	Follow-up	23.5 ± 2.2, 13–37	23.2 ± 3.0, 8–49
Serum IgG^c^	Baseline	11.1 ± 0.7, 7.5–18.3	10.7 ± 0.7, 5.4–15.6
	Follow-up	10.8 ± 0.7, 7.2–18.3	10.4 ± 0.6, 5.9–14.1
CSF-mononuclear cells, no × 10^6^/L, ref 0–5^c^	Baseline	0.9 ± 0.4, 0–4	1.6 ± 0.4, 0–4
	Follow-up	1.7 ± 0.6, 0–6	1.6 ± 0.6, 0–6
Neurofilament light (NFL) (ng/L)^c^	Baseline	276.9 ± 22.5, 170–450	289.3 ± 32.5, 160–640
	Follow-up	295.4 ± 23.1, 150–440	290.0 ± 36.7, 160–640

^a^One individual’s baseline sample in the ‘moderate-intensity’ exercise group was excluded because of a laboratory test failure regarding all results except of NFL and CSF mononuclear cells.

^b^Mean ± s.d., range.

^c^Mean ± s.e.m., range.

Reported adverse events were post-puncture headache after baseline CSF samplings in four participants of which two opted to remain in the study while two withdrew. The reported headaches were clinically typical of post-puncture headache, and in all cases self-limited with spontaneous resolution after a few days. No severe complications occurred.

### CSF and plasma protein immune-marker profiles

**Figure 2:**
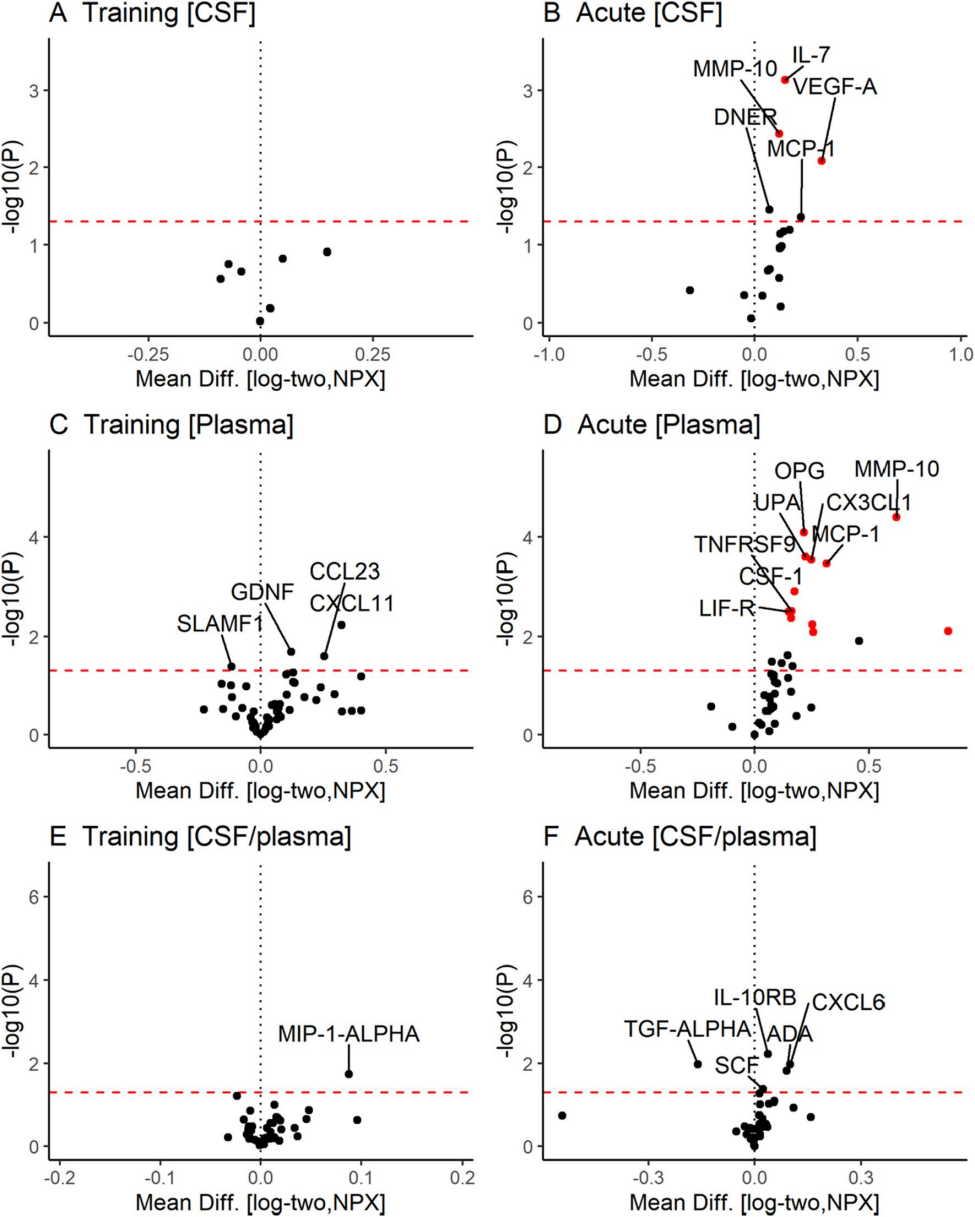
Changes in the immune marker profile of cerebrospinal fluid (CSF) and plasma after training and acute intervention exercise. Volcano plots summarize the mean difference and significance (P) from paired Student t-tests comparing baseline and follow-up levels of inflammation-related proteins after intervention with training [left] or acute [right] intensity exercise in CSF [first row], plasma [2nd row], and the ratio of CSF/plasma [3rd row]. The red dashed line indicates an exploratory cutoff of *P* = 0.05 and associations with P_FDR_ < 0.05 are highlighted red. Associations where filtered to exclude effects by sample handling variability (Fig. S3). R Core Team (2017). R: A language and environment for statistical computing. R Foundation for Statistical Computing, Vienna, Austria. URL https://www.R-project.org/.

A panel of 92 inflammation-related proteins was analyzed with PEA in both CSF and plasma. Using a false discovery threshold of 5% after adjustment for multiple comparisons and exclusion of low-stability proteins, we found a significant upregulation of 3 out of the 47 proteins in CSF after 4 days of acute physical activity, while none reached nominal significance after the training exercise intervention (Fig. 2A,B, Table 3) (Supplementary Table S1). In plasma, 12 out of 68 proteins were upregulated after the acute exercise program, while, again, none of the proteins reached nominal significance after the training exercise paradigm (Fig. 2C,D, Table 3) (Supplementary Table S2). Only proteins in CSF and plasma with a call rate of > 70% were included for analysis and were corrected for sample handling as displayed in Supplementary Table S3 and Supplementary Figure S1. In general, the degree of overlap between CSF and plasma protein profile was very low, since few proteins were found to be co-regulated in both compartments. We also performed a separate cross-sectional analysis of baseline protein levels in CSF and plasma in the two groups, which revealed only minor differences (Fig. 2E,F).

**Table 3:** Effects of anaerobic exercise on inflammation-related protein levels in cerebrospinal fluid and plasma.

	Training group				Acute group				Levels of intensity			
	Diff	*P*	P_cor_	P_FDR_	Diff	*P*	P_cor_	P_FDR_	β	*P*	P_cor_	P_FDR_
**Cerebrospinal fluid (CSF)**												
LAP-TGF-BETA-1	0.41	0.0063	0.027	0.84	0.82	1.00E−05	0.0065	0.1	0.39	2.20E−07	0.0012	0.056
UPA	0.03	0.68	0.5	0.99	0.1	0.22	0.0046	0.1	0.056	0.37	0.0073	0.11
TGF-ALPHA	− 0.072	0.17	0.21	0.99	− 0.051	0.44	0.0062	0.1	− 0.046	0.33	0.0035	0.081
HGF	− 0.002	0.96	0.42	0.99	0.11	0.16	0.046	0.31	0.035	0.53	0.022	0.17
MIP-1-ALPHA	0.23	0.0048	0.062	0.95	0.26	0.00026	0.07	0.4	0.14	0.00056	0.022	0.17
CXCL6	0.43	0.12	0.38	0.99	0.94	0.00058	0.019	0.17	0.49	1.00E−05	0.027	0.18
4E-BP1	0.22	0.0072	0.037	0.84	0.14	0.067	0.95	0.97	0.091	0.011	0.66	0.89
CD40	0.017	0.76	0.65	0.99	0.12	0.072	0.015	0.17	0.07	0.23	0.072	0.34
CX3CL1	− 0.019	0.84	0.18	0.99	0.22	0.058	0.029	0.22	0.079	0.14	0.022	0.17
**Plasma**												
IL-8	− 0.036	0.78	0.68	0.99	0.35	0.033	0.0033	0.073	0.13	0.094	0.0065	0.11
VEGF-A	0.1	0.15	0.29	0.99	0.21	0.0018	0.038	0.28	0.081	0.062	0.025	0.22
IL-7	0.12	0.31	0.48	0.99	0.28	0.079	0.046	0.28	0.073	0.52	0.15	0.47
OPG	0.13	0.084	0.71	0.99	0.21	8.10E−05	0.029	0.25	0.099	0.057	0.12	0.45
MCP-1	0.081	0.43	0.67	0.99	0.31	0.00034	0.0014	0.046	0.11	0.013	0.0095	0.13
CXCL1	0.38	0.28	0.08	0.99	0.64	0.035	0.015	0.17	0.27	0.069	0.0064	0.11
MMP-1	0.3	0.15	0.4	0.99	0.8	0.0025	0.014	0.17	0.37	0.021	0.0047	0.11
FGF-21	0.32	0.34	0.2	0.99	0.84	0.0079	0.042	0.28	0.26	0.14	0.11	0.45
IL-15RA	− 0.028	0.58	0.83	0.99	0.11	0.034	0.072	0.35	0.038	0.19	0.049	0.33
CXCL5	0.36	0.33	0.34	0.99	0.78	0.025	0.06	0.33	0.36	0.12	0.026	0.22
MMP-10	0.22	0.2	0.2	0.99	0.62	3.90E−05	0.00031	0.021	0.26	0.001	0.00016	0.011
DNER	0.045	0.25	0.1	0.99	0.074	0.033	0.35	0.58	0.056	0.13	0.043	0.32
CX3CL1	0.075	0.41	0.69	0.99	0.25	0.00029	0.014	0.17	0.073	0.076	0.086	0.41
CSF-1	0.027	0.61	0.85	0.99	0.17	0.0012	0.022	0.21	0.061	0.037	0.019	0.21

Summary statistics of paired Student *t* tests comparing before-and-after exercise intervention: mean difference (Diff) and significance (*P*). Linear regression model analyses correcting for sample handling variability was also examined (P_cor_, FDR corrected P_FDR_). Additional results are detailed in the supplementary material (Table S3).

### CSF and plasma neurotransmitter and kynurenine pathway metabolites

**Figure 3:**
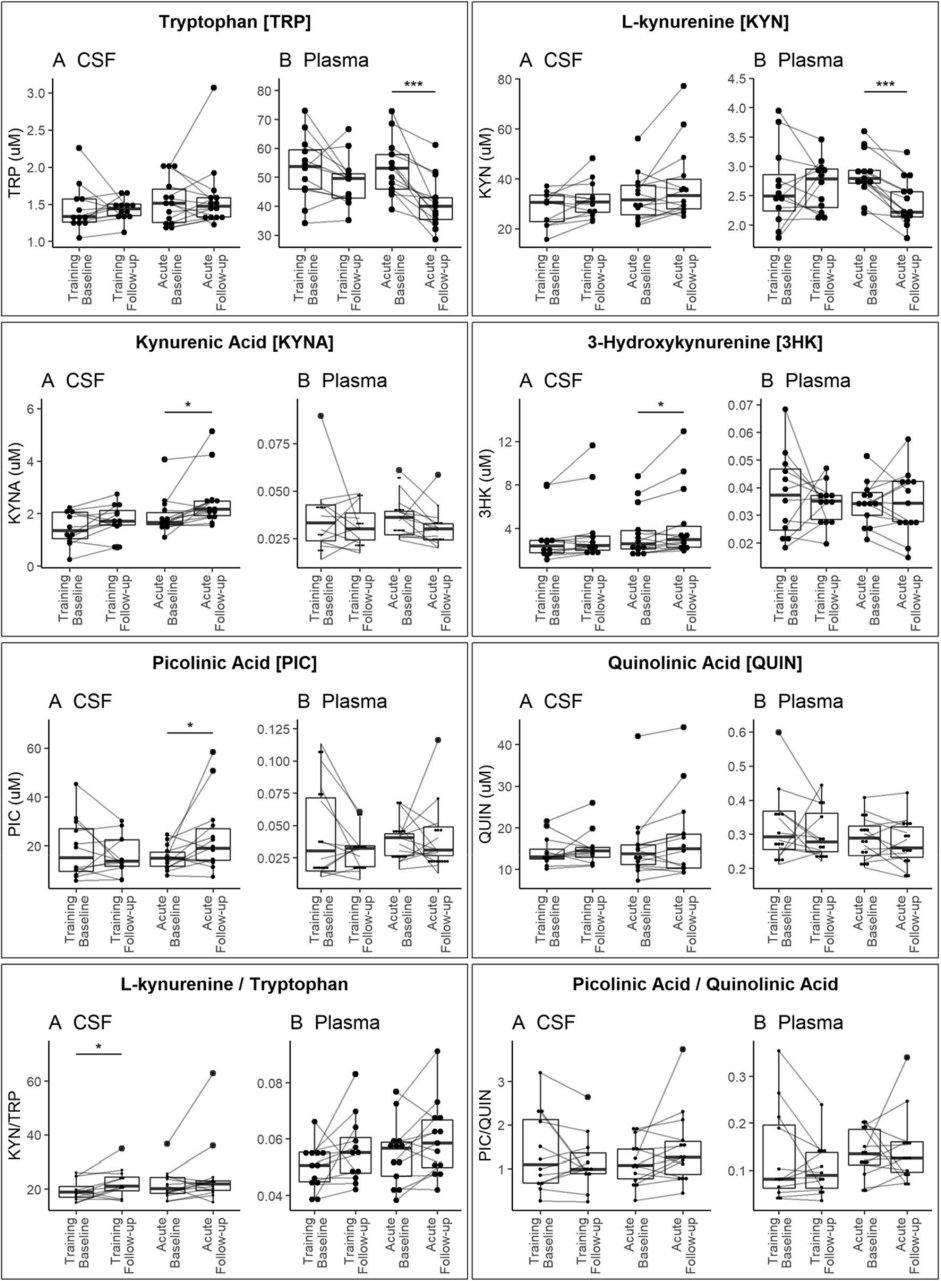
Distribution of tryptophan and kynurenine pathway metabolite levels in cerebrospinal fluid and plasma, stratified by intervention. Box and dot plots illustrate metabolite levels (μM) in (**A**) cerebrospinal fluid (CSF) and (**B**) plasma among paired samples (line) before and after either training or acute intensity aerobic exercise. Significance was determined across groups (solid bar). Significance levels (*P*): (*) < 0.05, (**) < 0.01, and (***) < 0.005. R: A language and environment for statistical computing. R Foundation for Statistical Computing, Vienna, Austria. URL https://www.R-project.org/.

The only conspicuous change in the concentrations of KP metabolites in plasma regarded a highly significant drop after exercise in the levels of the upstream components TRP and KYN in participants of the acute exercise protocol, while concentrations of the remaining metabolites remained stable (Fig. 3, Table 4). A different pattern was evident in the CSF, where concentrations of the downstream metabolites KYNA, 3HK and PIC increased after the acute exercise intervention, while TRP and KYN remained stable. In addition, the ratio of KYN/TRP, representing the first step in the KP, increased in the CSF of the training group. In contrast, there was only a trend for an increased ratio of PIC to QUIN, representing the neuroprotective and neurotoxic arm, respectively, of the downstream KP after acute exercise. As for the immune protein markers, correlations between concentrations of KP metabolites in plasma and CSF generally were weak (Supplementary Figure S2). However, interestingly PIC was an exception, showing a high degree of correlation between plasma and CSF levels both at baseline and follow up. As a consequence, also correlations between individual KP metabolites between CSF and plasma in general were weak, but here an interesting observation was that of an improved negative correlation between plasma levels of TRP and CSF levels of especially KYN, QUIN and PIC after exercise (Supplementary Figure S2).

**Table 4:** Effects of aerobic exercise on tryptophan and kynurenine pathway metabolite levels in cerebrospinal fluid and plasma.

	Training group				Acute group				Levels of intensity			
	Diff	*P*	P_cor_	P_FDR_	Diff	*P*	P_cor_	P_FDR_	β	*P*	P_cor_	P_FDR_
**Cerebrospinal fluid (CSF)**												
TRP	− 0.019	0.83	0.67	0.9	0.053	0.61	0.12	0.35	0.036	0.52	0.26	0.44
KYN	3.5	0.068	0.46	0.9	5.1	0.065	0.097	0.35	3.4	0.05	0.11	0.4
KYNA	0.3	0.068	0.43	0.9	0.57	0.017	0.29	0.47	0.37	0.0057	0.15	0.4
PIC	− 2.7	0.37	0.79	0.9	8	0.048	0.07	0.35	2.8	0.11	0.1	0.4
3HK	0.56	0.091	0.41	0.9	0.76	0.043	0.13	0.35	0.47	0.29	0.1	0.4
QUIN	0.81	0.44	0.78	0.9	2	0.087	0.19	0.35	1.2	0.33	0.21	0.44
GLU	− 0.015	0.87	0.85	0.9	0.093	0.24	0.17	0.35	0.034	0.39	0.28	0.44
GABA	− 0.34	0.59	0.83	0.9	− 0.55	0.27	0.81	0.81	− 0.041	0.87	0.74	0.77
SERINE	− 0.19	0.81	0.9	0.9	1.4	0.036	0.062	0.35	0.55	0.11	0.15	0.4
KYNA-2	0.27	0.22	0.57	0.9	0.37	0.074	0.69	0.78	0.24	0.033	0.5	0.61
KYN/TRP	2.7	0.038	0.26	0.9	3.3	0.15	0.44	0.64	2	0.093	0.31	0.44
KYNA/TRP	0.24	0.082	0.3	0.9	0.27	0.051	0.72	0.78	0.21	0.023	0.77	0.77
PIC/QUIN	− 0.23	0.24	0.77	0.9	0.31	0.12	0.5	0.64	0.082	0.5	0.52	0.61
**Plasma**												
TRP	− 4.3	0.15	0.35	0.75	− 12	0.00029	7.50E−05	0.00082	− 5.7	1.30E−05	6.60E−05	0.00073
KYN	0.036	0.84	0.32	0.75	− 0.46	1.10E−05	0.0059	0.033	− 0.17	0.039	0.072	0.26
KYNA	− 0.0049	0.42	0.48	0.75	− 0.0056	0.11	0.18	0.56	− 0.0028	0.18	0.19	0.41
PIC	− 0.013	0.12	0.33	0.75	0.0018	0.83	0.95	0.99	− 0.00097	0.82	0.82	0.9
3HK	− 0.0032	0.5	0.71	0.83	− 7.50E−05	0.98	0.99	0.99	− 0.00083	0.65	0.69	0.85
QUIN	− 0.022	0.5	0.78	0.83	− 0.014	0.32	0.72	0.99	− 0.015	0.28	0.65	0.85
NAM	− 0.014	0.61	0.45	0.75	0.026	0.47	0.33	0.61	0.0025	0.92	0.32	0.58
XA	− 0.0026	0.65	0.7	0.83	− 0.0078	0.1	0.21	0.56	− 0.0038	0.088	0.14	0.39
KYN/TRP	0.006	0.11	0.058	0.63	0.0049	0.066	0.33	0.61	0.0035	0.055	0.068	0.26
KYNA/TRP	− 4.90E−06	0.96	0.83	0.83	6.50E−05	0.35	0.53	0.83	3.70E−05	0.38	0.54	0.84
PIC/QUIN	− 0.028	0.18	0.25	0.75	0.0074	0.75	0.94	0.99	0.0029	0.82	0.92	0.92

Summary statistics of paired Student t-tests comparing before-and-after exercise intervention: mean difference (Diff) and significance (*P*). Linear regression model analyses correcting for sample handling variability was also examined (P_cor_, FDR corrected P_FDR_).

**Figure 4:**
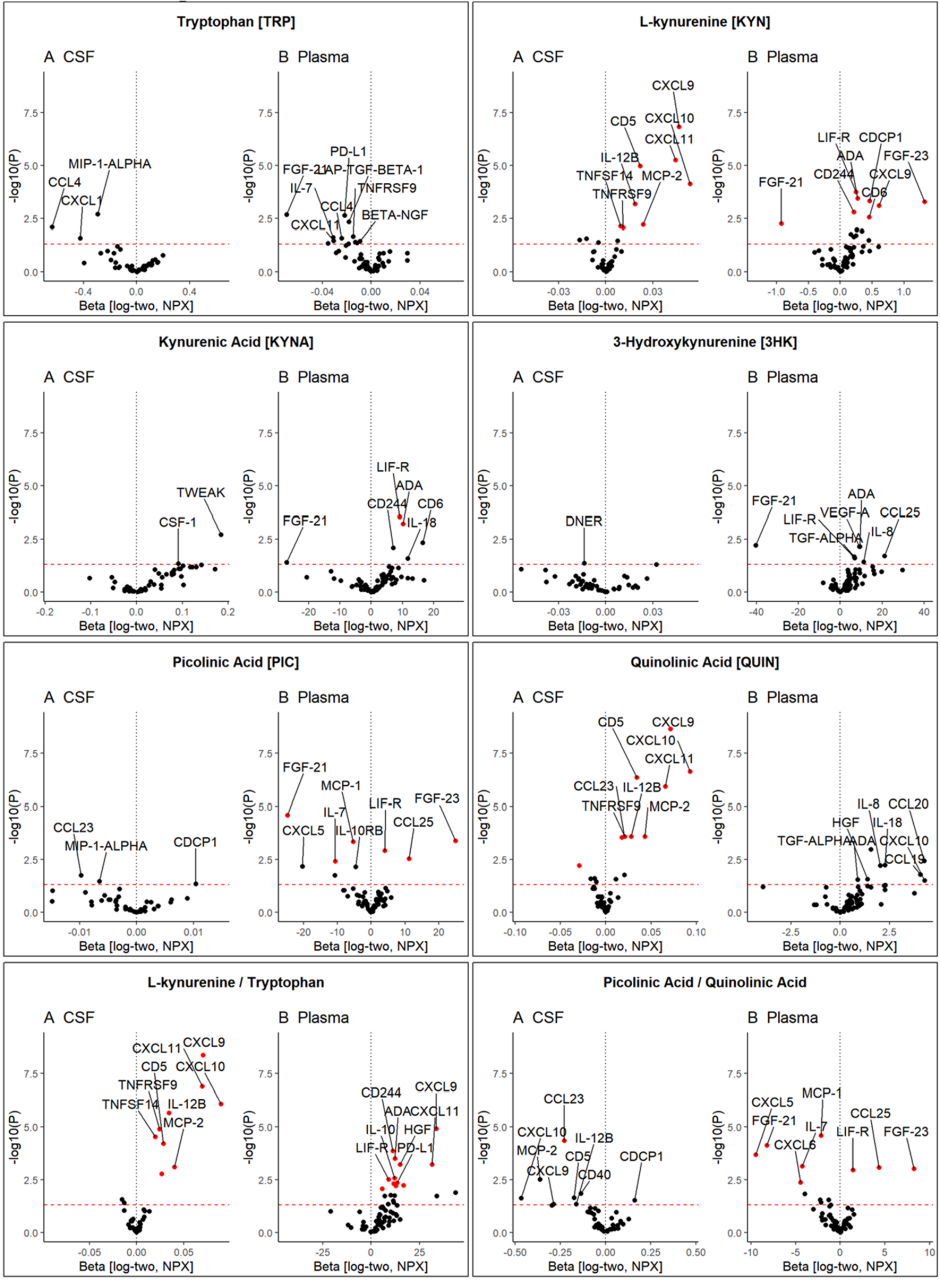
Correlation between tryptophan and kynurenine pathway metabolites and immune-related protein levels. Volcano plots summarize the effect size (B) and significance (*P*) from linear regression analyses between metabolite and protein levels adjusted for age at baseline, sex, intervention, and sample handling variability. Red dashed line indicates an exploratory cutoff of *P* = 0.05 and associations with P_FDR_ < 0.05 are highlighted red. R: A language and environment for statistical computing. R Foundation for Statistical Computing, Vienna, Austria. URL https://www.R-project.org/.

In order to study if changes in immune marker profiles in CSF and plasma after physical exercise were associated with changes in KP metabolites, we performed correlations between the two combining the two exercise groups. Plasma levels of KYN and PIC, and to a lesser degree KYNA, were associated with changes in protein immune markers (Fig. 4). In addition, both the ratios of KYN to TRP and PIC to QUIN were also associated with an altered immune marker profile. In the CSF, levels of KYN and QUIN, as well as the ratio of KYN to TRP and, to a lesser degree, PIC to QUIN were similarly associated with immune protein marker profiles (Fig. 4).

Concentrations of the neurotransmitters GLU and GABA pre-/post-intervention were unchanged in both groups, while levels of serine increased after acute exercise (Supplementary Figure S3).

## Discussion

To date data on the effect of physical exercise on immune protein marker and KP metabolite profiles assessed with paired sampling in blood and CSF in healthy subjects are lacking. Our main findings are that while changes in immune protein markers were modest, changes in KP metabolites were more conspicuous. There was also evidence of co-regulation, since levels of several immune protein markers and KP metabolites correlated both in plasma and in CSF. Furthermore, changes almost exclusively were detected in the acute exercise group, where the participants performed acute bouts of exercise over four consecutive days, while changes induced by engaging in three times weekly training exercise over 4 weeks, led to very limited effects. A further observation was that the correlation between plasma and CSF concentrations of the studied markers was low, indicating limited equilibration through an intact blood brain barrier (BBB). The observation of limited correlation between plasma and CSF also held true for KP metabolites. However, with the exception of PIC, that correlated to a high degree between compartments both before and after the intervention.

One of the proteins being upregulated in the CSF after the acute exercise intervention was vascular endothelial growth factor (VEGF). VEGF has been observed to increase in blood proportionally to the intensity of exercise, which is consistent with our finding in CSF^30^. The only protein being upregulated after acute exercise in both plasma and CSF was matrix metalloproteinase (MMP)-10, which belong to a group of endopeptidases mainly responsible for degrading extracellular matrix proteins necessary for wound healing and vascular remodeling. While increased levels of MMP-2 and -9 in blood have been associated with high intensity exercise, increases in MMP-10 in both the peripheral and central compartment is a novel finding, but functionally consistent with increases in VEGF and osteoprotegerin^31^. Several growth factors of different families were increased in the acute group including IL-7 in the CSF, transforming growth factor alpha, delta and notch-like epidermal growth factor and leukemia inhibitory factor receptor in plasma, none of which previously have been associated with exercise, in contrast to FGF-21, which is a well described myokine^4^. Also, several chemokines and cytokines were increased in the plasma of the acute group, some of which are related to innate immune functions, e.g. monocyte chemoattractant protein 1 (MCP-1 or CCL2), chemokine (C-X3-C motif) ligand 1 (CX3CL1) and colony stimulating factor 1. MCP-1 also displayed a trend for being increased in the CSF. A study in mice has shown that muscle activation induces lymphocyte traffic across the BBB, mainly mediated by IL-6 induced expression of CCL20^32^. Here we found a trend for increased levels of CCL20 in plasma after acute exercise, but not CSF and there was no indication that the concentrations of mononuclear cells increased after exercise in the intrathecal compartment.

Based on findings in prior studies, a number of cytokines were expected to occur in measurable levels and increase in response to the interventions. These include IL-6, a central myokine associated with muscle growth and regulation of energy metabolism, the newly discovered IL-13, necessary for development of muscle endurance with exercise, brain-derived neurotrophic factor (BDNF), which has been observed to increase in blood proportionally to exercise intensity, and interferon (IFN)-γ/α, necessary for induction of indoleamine 2,3-dioxygenase (IDO) as part of the KP^30,33–35^. It is likely that the inability to reliably detect these proteins here depend on technical issues, such as for example the performance of the antibodies used for detection and that other assay platforms might display improved sensitivity for these particular targets.

Next, we determined the CSF and plasma concentrations of KP metabolites and classical neurotransmitters with HPLC or LC/MS. Although prior studies have assessed dynamic changes of the KP in the peripheral compartment, the heterogeneity of the studies precludes more detailed knowledge relating to outcomes on KP metabolites^18^. Evidence relating to physical exercise changes to the KP pattern in humans in the intrathecal compartment, with paired sampling also from the peripheral compartment has been lacking. From our results, we only found evidence of changes in KP metabolites in the plasma of participants of the acute protocol. This is in line with the current evidence, where acute bouts of exercise induces dynamic changes of the KP, rather than long-term modifications of KP metabolites from exercise^18^. While concentrations of TRP and KYN demonstrated a clear drop in plasma after exercise, the downstream metabolites KYNA, 3-HK and PIC increased in the CSF. Still, the correlation between plasma and CSF levels were weak, suggesting that these changes reflect different modalities of activation of the KP in the respective compartment. An interesting observation, however, was that the reduction in TRP in plasma resulting from exercise increased the strength of a negative correlation with CSF levels of KYN, QUIN and PIC. In the study by Agudelo and co-workers, addressing resilience to stress-induced depressive behavior in mice, activity of PGC1α was found to increase the conversion of KYN to KYNA in the periphery, resulting in less influx of KYN to the brain since KYNA unlike KYN has a poor BBB penetrance^9,36^. In these mice they also reported a significant correlation between plasma KYN and brain 3HK levels, but not with KYNA. This contrasts with our findings here, where plasma levels of KYN both before and after exercise correlated poorly with 3HK. However, also here, albeit weaklier, plasma KYN levels correlated negatively with QUIN and KYNA after exercise. It cannot be expected that findings obtained in a transgenic mouse model can be directly translated to man, however, our results corroborate the notion that muscle activity leads to reduced levels of TRP and KYN in blood and that this correlate with alterations of the KP in brain. Also, it is of interest that exercise induced increases of KYNA and PIC, which both have been ascribed neuroprotective activities, while only levels of potentially neurotoxic 3HK, but not QUIN increased^10^. There was also a trend for an increased ratio of PIC to QUIN, which represents the neuroprotective versus neurotoxic arm of the downstream KP. Even if we did not directly address activity of IDO and tryptophan 2,3-dioxygenase 2 (TDO2) enzyme activity, known to be regulated by cytokines such as IFN-γ and IL-1β, the fact that levels of different KP metabolites correlated to immune protein profiles suggest a degree of co-regulation, in line with previous notions of immune regulation of the KP^37–39^. In contrast, concentrations of classical neurotransmitters, i.e. GLU and GABA, remained stable in the CSF, while serine was found to be increased with the acute protocol.

In context of previous neuropsychiatric research, the following things are worth highlighting. First, low levels of VEGF in blood and CSF, have been of particular interest in regards to treatment resistant depression^40–42^, as well as suicidal behavior^43,44^. Second, the d-isomer of serine has been of interest as potential biomarker and therapeutic agent for patients with major depressive disorder and schizophrenia^45^, both of which also benefiting from exercise therapy^2^.

It is well accepted that physical exercise can lead to changes in cytokine levels and KP metabolites in the peripheral compartment. It has however been unknown to what degree such data can be extrapolated to the intrathecal compartment. This likely depends partly on the fact that collection of CSF by lumbar puncture is considered an invasive procedure associated with post-puncture headache in up to a third of cases^46^. However, this risk can be significantly reduced by the use of atraumatic (blunt) needles^47,48^. In a large cohort of elective research lumbar punctures, headache developed in 5.6%, of which 92% were mild and resolved spontaneously^49^. Our results are largely in agreement with this study, since we recorded 4 (7.1%) cases of mild post-puncture headache over 56 lumbar punctures, all of which were mild and resolved spontaneously. Thus, the study protocol applied proved to be safe, and with acceptable levels of post-puncture symptoms. The general lack of correlation between the compartments is interesting, and in line with previous biomarker studies, such as studies looking at suicidal behavior^50,51^, as well as a study using the same assay methodology^52^. This would suggest that there are differences in immune marker expression as an effect from exercise, and plausibly different physiological roles in the peripheral versus the intrathecal compartment.

A strength of this study is the access to paired samples before and after intervention, which limits the risk of confounders. Even though the sample size is small, this is by far the largest study having access to paired samples in blood and CSF, both before and after exercise intervention. Furthermore, this is the first study assessing immune markers, and KP metabolites from both compartments, pre- and post, two different exercise paradigms. We analyzed paired samples with a novel high-throughput multiplex proteomic technical platform that combines probe-linked antibody detection with a polymerase chain reaction, which provides a higher sensitivity while retaining a high degree of reproducibility compared to many other available detection systems^26^. It is, however, important to be aware that since very low concentrations of proteins can be detected, it is unknown if changes in individual proteins will be of biological significance. Nevertheless, the detection of changes in a larger set of proteins obtained from paired samples collected under stringent conditions using a highly reproducible technique is ideal to address the study aim, i.e. to explore if physical exercise changes immune marker profiles in plasma and CSF. Accordingly, we analyzed samples with an inflammation panel that has been used in many previous studies^52–55^.

A few limitations need highlighting. The rationale behind the two exercise paradigms, was to compare differences in dynamic changes from more vigorous bouts of frequent exercise, compared to effects from more moderate exercise over time, also based on the previous literature. The choice of different exercise modalities has its limitations though, since groups could be expected not to be fully equal in relation to previous exercise habits, meaning that we cannot preclude that other unknown variables are at hand, explaining some of the differences between the groups. In relation to this, the limited effects reported in the training group also could reflect the small sample size that would exempt detection of subtler dynamic changes. Furthermore, another important limitation applied within the study design was the lack of a control group for both paradigms, and that the exercise intervention was performed without objective supervision of adherence and intensity. However, all subjects completing the trial provided detailed information on quality and quantity of all training sessions, supporting the notion that the intervention was carried out as instructed. A further limitation is that we only used self-reported exercise habits to allocate participants rather than objective physiological testing of their physical fitness. However, we could not detect significant differences between the two groups in the baseline levels of the analyzed biomarkers. Furthermore, by way of the design of the study, the time span between the two lumbar punctures differed between the two groups. Therefore, we cannot exclude that this may represent a confounder for some of the findings.

In conclusion, we here observed changes in KP metabolites and also, to a lesser degree, immune marker profiles in blood and the CSF after an acute vigorous aerobic exercise intervention, which contrasted to findings for the training exercise protocol over a longer time frame. The observed dynamic changes of immune markers may have relevance for the beneficial clinical effects of physical exercise previously demonstrated across a number of conditions, including several psychiatric and neurological diseases, but also suggest that the existence of a dose–response relationship, i.e. that more acute bouts of exercise might be needed to result in significant changes of markers, and plausibly long-term modifications and homeostasis on relevant enzyme activities. Further studies are needed to address the durability of effects and also if other modalities of exercise, such as muscle strength training, may display similar or different results.

## Supplementary Information

Supplementary Information
