# Supplementary material for: Differential effects on blood and cerebrospinal fluid immune protein markers and kynurenine pathway metabolites from aerobic physical exercise in healthy subjects
Source: Sci Rep. 2021 Jan 18;11:1669. doi: 10.1038/s41598-021-81306-4 (PMC7814004; doi:10.1038/s41598-021-81306-4)
Supplement: Supplementary file 1 — Supplementary Information [file 41598_2021_81306_MOESM1_ESM.docx]

## Differential effects on blood and cerebrospinal fluid immune protein markers and kynurenine pathway metabolites from aerobic physical exercise in healthy subjects

Josef Isung^1,*,^**^⸸^**, Mathias Granqvist^2,^**^⸸^**, Ada Trepci^3^, Jesse Huang^2^, Lilly Schwieler^3^, Marie Kierkegaard^4^, Sophie Erhardt^3^, Jussi Jokinen^1, 5^, and Fredrik Piehl^2^

^1^Centre for Psychiatry Research, Department of Clinical Neuroscience, Karolinska Institutet, & Stockholm Health Care Services, Region Stockholm, Stockholm, Sweden

^2^Division of Physiotherapy, Division of Neurology, Department of Clinical Neuroscience, Karolinska Institutet, & Academic Specialist Center, Stockholm Health Care Services, Region Stockholm, Stockholm, Sweden

^3^Department of Physiology and Pharmacology, Karolinska Institutet, Stockholm, Sweden.

^4^Department of Neurobiology, Care Sciences and Society, Karolinska Institutet & Academic Specialist Center, Stockholm Health Care Services, Region Stockholm, Stockholm, Sweden.

^5^Department of Clinical Sciences, Umeå University, Umeå, Sweden

^*^corresponding author: josef.isung@ki.se

^⸸^these authors contributed equally to this work

**Supplementary Tables and Figures**

| **Table S1.** Effects of aerobic exercise on inflammation-related protein levels in cerebrospinal fluid (continued). |
| --- |
| **Table S2.** Effects of aerobic exercise on inflammation-related protein levels in plasma (continued). |
| **Table S3.** Call rate of proteins measured by the Olink Proseek Inflammation I^96x96^ panel for cerebrospinal fluid and plasma. |
| **Figure S1.** Effects of aerobic exercise on inflammation-related protein levels in cerebrospinal fluid and plasma correcting for variation in sample handling. |
| **Figure S2**. Correlation between cerebrospinal fluid and plasma metabolites. |
| **Figure S3.** Distribution of glutamate, GABA, and serine in cerebrospinal fluid, stratified by intervention group and sampling time. |

**Table S1.** Effects of aerobic exercise on inflammation-related protein levels in cerebrospinal fluid (continued).

|  | **Training Intervention Group** | | | | **Acute Intervention Group** | | | | **Levels of Intensity** | | | |
| --- | --- | --- | --- | --- | --- | --- | --- | --- | --- | --- | --- | --- |
|  | **Diff** | **P** | **P_cor_** | **P_FDR_** | **Diff** | **P** | **P_cor_** | **P_FDR_** | **β** | **P** | **P_cor_** | **P_FDR_** |
| IL-8 | 0.24 | 0.042 | 0.53 | 0.99 | 0.32 | 0.016 | 0.38 | 0.79 | 0.17 | 0.02 | 0.52 | 0.88 |
| VEGF-A | 0.26 | 0.015 | 0.34 | 0.99 | 0.32 | 0.0083 | 0.32 | 0.79 | 0.16 | 0.035 | 0.66 | 0.89 |
| CDCP1 | 0.15 | 0.12 | 0.84 | 0.99 | 0.28 | 0.0017 | 0.62 | 0.88 | 0.11 | 0.1 | 0.49 | 0.87 |
| IL-7 | 0.11 | 0.12 | 0.17 | 0.99 | 0.15 | 0.00073 | 0.12 | 0.52 | 0.066 | 0.016 | 0.062 | 0.34 |
| OPG | 0.045 | 0.62 | 0.64 | 0.99 | 0.2 | 0.027 | 0.65 | 0.88 | 0.086 | 0.19 | 0.33 | 0.72 |
| LAP-TGF-BETA-1 | 0.41 | 0.0063 | 0.027 | 0.84 | 0.82 | 1.00E-05 | 0.0065 | 0.1 | 0.39 | 2.20E-07 | 0.0012 | 0.056 |
| UPA | 0.03 | 0.68 | 0.5 | 0.99 | 0.1 | 0.22 | 0.0046 | 0.1 | 0.056 | 0.37 | 0.0073 | 0.11 |
| MCP-1 | 0.014 | 0.79 | 0.66 | 0.99 | 0.23 | 0.044 | 0.41 | 0.8 | 0.089 | 0.043 | 0.54 | 0.88 |
| CXCL11 | 0.18 | 0.21 | 0.81 | 0.99 | 0.34 | 0.0054 | 0.29 | 0.79 | 0.19 | 0.15 | 0.67 | 0.89 |
| TRAIL | 0.096 | 0.18 | 0.65 | 0.99 | 0.12 | 0.26 | 0.8 | 0.92 | 0.081 | 0.045 | 0.78 | 0.9 |
| CCL4 | 0.15 | 0.24 | 0.66 | 0.99 | 0.3 | 0.012 | 0.72 | 0.9 | 0.073 | 0.43 | 0.99 | 0.99 |
| SCF | 0.12 | 0.066 | 0.91 | 0.99 | 0.28 | 0.0049 | 0.089 | 0.45 | 0.12 | 0.061 | 0.13 | 0.46 |
| IL-18 | 0.098 | 0.14 | 0.48 | 0.99 | 0.12 | 0.11 | 0.52 | 0.88 | 0.066 | 0.016 | 0.45 | 0.87 |
| TGF-ALPHA | -0.072 | 0.17 | 0.21 | 0.99 | -0.051 | 0.44 | 0.0062 | 0.1 | -0.046 | 0.33 | 0.0035 | 0.081 |
| CCL11 | 0.1 | 0.26 | 0.35 | 0.99 | 0.16 | 0.0063 | 0.23 | 0.69 | 0.069 | 0.024 | 0.32 | 0.72 |
| FGF-5 | 0.0029 | 0.95 | 0.34 | 0.99 | 0.16 | 0.048 | 0.33 | 0.79 | 0.044 | 0.47 | 0.17 | 0.46 |
| MMP-1 | 0.12 | 0.26 | 0.62 | 0.99 | 0.29 | 0.013 | 0.85 | 0.93 | 0.15 | 0.089 | 0.71 | 0.89 |
| LIF-R | 0.054 | 0.4 | 0.66 | 0.99 | 0.28 | 0.0041 | 0.44 | 0.8 | 0.1 | 0.077 | 0.28 | 0.71 |
| CCL19 | 0.38 | 0.25 | NA | NA | 1.2 | 9.80E-05 | NA | NA | 0.64 | 2.70E-05 | NA | NA |
| IL-10RB | 0.097 | 0.078 | 0.82 | 0.99 | 0.25 | 0.002 | 0.59 | 0.88 | 0.1 | 0.032 | 0.44 | 0.87 |
| IL-18R1 | 0.024 | 0.59 | 0.79 | 0.99 | 0.12 | 0.036 | 0.15 | 0.53 | 0.03 | 0.67 | 0.15 | 0.46 |
| PD-L1 | 0.14 | 0.11 | 0.92 | 0.99 | 0.28 | 0.0022 | 0.71 | 0.9 | 0.13 | 0.033 | 0.38 | 0.8 |
| BETA-NGF | 0.086 | 0.14 | 0.86 | 0.99 | 0.21 | 0.00062 | 0.59 | 0.88 | 0.092 | 0.012 | 0.61 | 0.89 |
| HGF | -0.002 | 0.96 | 0.42 | 0.99 | 0.11 | 0.16 | 0.046 | 0.31 | 0.035 | 0.53 | 0.022 | 0.17 |
| MMP-10 | 0.022 | 0.66 | 0.88 | 0.99 | 0.12 | 0.0037 | 0.62 | 0.88 | 0.048 | 0.043 | 0.75 | 0.9 |
| MIP-1-ALPHA | 0.23 | 0.0048 | 0.062 | 0.95 | 0.26 | 0.00026 | 0.07 | 0.4 | 0.14 | 0.00056 | 0.022 | 0.17 |
| CXCL6 | 0.43 | 0.12 | 0.38 | 0.99 | 0.94 | 0.00058 | 0.019 | 0.17 | 0.49 | 1.00E-05 | 0.027 | 0.18 |
| CXCL10 | 0.31 | 0.11 | 0.99 | 0.99 | 0.43 | 0.028 | 0.36 | 0.79 | 0.27 | 0.12 | 0.59 | 0.89 |
| 4E-BP1 | 0.22 | 0.0072 | 0.037 | 0.84 | 0.14 | 0.067 | 0.95 | 0.97 | 0.091 | 0.011 | 0.66 | 0.89 |
| DNER | -0.0015 | 0.95 | 0.32 | 0.99 | 0.072 | 0.035 | 0.22 | 0.69 | 0.024 | 0.31 | 0.13 | 0.46 |
| CD40 | 0.017 | 0.76 | 0.65 | 0.99 | 0.12 | 0.072 | 0.015 | 0.17 | 0.07 | 0.23 | 0.072 | 0.34 |
| MCP-2 | 0.22 | 0.044 | 0.95 | 0.99 | 0.31 | 0.0048 | 0.88 | 0.94 | 0.16 | 0.14 | 0.94 | 0.98 |
| CX3CL1 | -0.019 | 0.84 | 0.18 | 0.99 | 0.22 | 0.058 | 0.029 | 0.22 | 0.079 | 0.14 | 0.022 | 0.17 |
| TNFRSF9 | -0.03 | 0.67 | 0.4 | 0.99 | 0.14 | 0.015 | 0.61 | 0.88 | 0.082 | 0.14 | 0.56 | 0.89 |
| TWEAK | 0.06 | 0.39 | 0.46 | 0.99 | 0.28 | 0.0069 | 0.37 | 0.79 | 0.1 | 0.12 | 0.15 | 0.46 |
| ADA | 0.11 | 0.087 | 0.82 | 0.99 | 0.39 | 4.00E-04 | 0.92 | 0.96 | 0.14 | 0.051 | 0.81 | 0.9 |

**Table S2.** Effects of aerobic exercise on inflammation-related protein levels in plasma (continued).

|  | **Training Intervention Group** | | | | **Acute Intervention Group** | | | | **Levels of Intensity** | | | |
| --- | --- | --- | --- | --- | --- | --- | --- | --- | --- | --- | --- | --- |
|  | **Diff** | **P** | **P_cor_** | **P_FDR_** | **Diff** | **P** | **P_cor_** | **P_FDR_** | **β** | **P** | **P_cor_** | **P_FDR_** |
| IL-8 | -0.036 | 0.78 | 0.68 | 0.99 | 0.35 | 0.033 | 0.0033 | 0.073 | 0.13 | 0.094 | 0.0065 | 0.11 |
| VEGF-A | 0.1 | 0.15 | 0.29 | 0.99 | 0.21 | 0.0018 | 0.038 | 0.28 | 0.081 | 0.062 | 0.025 | 0.22 |
| GDNF | 0.12 | 0.021 | 0.15 | 0.99 | 0.064 | 0.17 | 0.34 | 0.58 | 0.012 | 0.72 | 0.6 | 0.74 |
| IL-7 | 0.12 | 0.31 | 0.48 | 0.99 | 0.28 | 0.079 | 0.046 | 0.28 | 0.073 | 0.52 | 0.15 | 0.47 |
| OPG | 0.13 | 0.084 | 0.71 | 0.99 | 0.21 | 8.10E-05 | 0.029 | 0.25 | 0.099 | 0.057 | 0.12 | 0.45 |
| UPA | 0.032 | 0.68 | 0.91 | 0.99 | 0.22 | 0.00025 | 0.13 | 0.4 | 0.11 | 0.061 | 0.086 | 0.41 |
| MCP-1 | 0.081 | 0.43 | 0.67 | 0.99 | 0.31 | 0.00034 | 0.0014 | 0.046 | 0.11 | 0.013 | 0.0095 | 0.13 |
| CXCL11 | 0.32 | 0.006 | 0.34 | 0.99 | 0.2 | 0.23 | 0.32 | 0.57 | 0.12 | 0.29 | 0.21 | 0.47 |
| AXIN1 | -1 | 0.027 | NA | NA | -0.23 | 0.51 | NA | NA | -0.32 | 0.053 | NA | NA |
| CXCL1 | 0.38 | 0.28 | 0.08 | 0.99 | 0.64 | 0.035 | 0.015 | 0.17 | 0.27 | 0.069 | 0.0064 | 0.11 |
| CCL4 | 0.068 | 0.32 | 0.63 | 0.99 | 0.26 | 0.0082 | 0.15 | 0.42 | 0.06 | 0.48 | 0.31 | 0.5 |
| SCF | 0.057 | 0.24 | 0.9 | 0.99 | 0.12 | 0.035 | 0.38 | 0.6 | 0.02 | 0.69 | 0.9 | 0.93 |
| IL-18 | -0.021 | 0.67 | 0.92 | 0.99 | 0.14 | 0.024 | 0.35 | 0.58 | 0.056 | 0.45 | 0.29 | 0.49 |
| SLAMF1 | -0.12 | 0.041 | 0.82 | 0.99 | 0.073 | 0.3 | 0.55 | 0.7 | 0.0069 | 0.93 | 0.41 | 0.59 |
| TGF-ALPHA | -0.011 | 0.86 | 0.94 | 0.99 | 0.16 | 0.0042 | 0.09 | 0.35 | 0.049 | 0.15 | 0.14 | 0.47 |
| MCP-4 | 0.13 | 0.055 | 0.64 | 0.99 | 0.2 | 0.018 | 0.2 | 0.47 | 0.097 | 0.26 | 0.15 | 0.47 |
| CCL11 | 0.012 | 0.87 | 0.62 | 0.99 | 0.25 | 0.0058 | 0.12 | 0.4 | 0.064 | 0.38 | 0.23 | 0.47 |
| MMP-1 | 0.3 | 0.15 | 0.4 | 0.99 | 0.8 | 0.0025 | 0.014 | 0.17 | 0.37 | 0.021 | 0.0047 | 0.11 |
| LIF-R | -0.035 | 0.54 | 0.68 | 0.99 | 0.15 | 0.0032 | 0.098 | 0.35 | 0.049 | 0.18 | 0.24 | 0.47 |
| FGF-21 | 0.32 | 0.34 | 0.2 | 0.99 | 0.84 | 0.0079 | 0.042 | 0.28 | 0.26 | 0.14 | 0.11 | 0.45 |
| IL-15RA | -0.028 | 0.58 | 0.83 | 0.99 | 0.11 | 0.034 | 0.072 | 0.35 | 0.038 | 0.19 | 0.049 | 0.33 |
| CXCL5 | 0.36 | 0.33 | 0.34 | 0.99 | 0.78 | 0.025 | 0.06 | 0.33 | 0.36 | 0.12 | 0.026 | 0.22 |
| HGF | 0.028 | 0.55 | 0.89 | 0.99 | 0.16 | 0.04 | 0.22 | 0.47 | 0.029 | 0.59 | 0.47 | 0.65 |
| MMP-10 | 0.22 | 0.2 | 0.2 | 0.99 | 0.62 | 3.90E-05 | 0.00031 | 0.021 | 0.26 | 0.001 | 0.00016 | 0.011 |
| CCL23 | 0.25 | 0.025 | 0.92 | 0.99 | 0.15 | 0.037 | 0.35 | 0.58 | 0.085 | 0.27 | 0.48 | 0.65 |
| FLT3L | 0.064 | 0.49 | 0.51 | 0.99 | 0.12 | 0.049 | 0.087 | 0.35 | 0.046 | 0.3 | 0.18 | 0.47 |
| SIRT2 | -0.77 | 0.032 | 0.87 | 0.99 | -0.041 | 0.9 | 0.29 | 0.57 | -0.19 | 0.19 | 0.17 | 0.47 |
| DNER | 0.045 | 0.25 | 0.1 | 0.99 | 0.074 | 0.033 | 0.35 | 0.58 | 0.056 | 0.13 | 0.043 | 0.32 |
| MCP-2 | 0.1 | 0.06 | 0.65 | 0.99 | 0.21 | 0.04 | 0.43 | 0.63 | 0.076 | 0.57 | 0.61 | 0.75 |
| CCL25 | 0.076 | 0.24 | 0.28 | 0.99 | 0.19 | 0.014 | 0.57 | 0.71 | 0.099 | 0.35 | 0.27 | 0.48 |
| CX3CL1 | 0.075 | 0.41 | 0.69 | 0.99 | 0.25 | 0.00029 | 0.014 | 0.17 | 0.073 | 0.076 | 0.086 | 0.41 |
| TNFRSF9 | 0.02 | 0.7 | 0.83 | 0.99 | 0.16 | 0.0031 | 0.21 | 0.47 | 0.051 | 0.35 | 0.19 | 0.47 |
| CCL20 | -0.23 | 0.31 | 0.96 | 0.99 | 0.46 | 0.013 | 0.13 | 0.4 | 0.12 | 0.37 | 0.16 | 0.47 |
| STAMPB | -0.57 | 0.02 | 0.87 | 0.99 | 0.035 | 0.89 | 0.098 | 0.35 | -0.12 | 0.25 | 0.06 | 0.37 |
| CSF-1 | 0.027 | 0.61 | 0.85 | 0.99 | 0.17 | 0.0012 | 0.022 | 0.21 | 0.061 | 0.037 | 0.019 | 0.21 |

**Table S3.** Call rate of proteins measured by the Olink Proseek Inflammation I^96x96^ panel for cerebrospinal fluid (CSF) and plasma.

| **Symbol** | **Protein name** | **Call rate (%)** | |
| --- | --- | --- | --- |
|  |  | **CSF** | **Plasma** |
| ADA | Adenosine Deaminase | **>70%** | **>70%** |
| ARTN | Artemin | **<30%** | **<30%** |
| AXIN1 | Axin-1 | **<30%** | **>70%** |
| Beta-NGF | β-nerve growth factor | **>70%** | **>70%** |
| BDNF | Brain-derived neurotrophic factor | **-** | **-** |
| CASP-8 | Caspase 8 | **<30%** | **>70%** |
| CCL19 | C-C motif chemokine 19 | **>70%** | **>70%** |
| CCL20 | C-C motif chemokine 20 | **<30%** | **>70%** |
| CCL23 | C-C motif chemokine 23 | **>70%** | **>70%** |
| CCL25 | C-C motif chemokine 25 | **30-70%** | **>70%** |
| CCL28 | C-C motif chemokine 28 | **<30%** | **>70%** |
| CCL3/MIP-1a | C-C motif chemokine 3 | **>70%** | **>70%** |
| CCL4 | C-C motif chemokine 4 | **>70%** | **>70%** |
| CD40 | CD40L receptor | **>70%** | **>70%** |
| CDCP1 | CUB domain-containing protein 1 | **>70%** | **>70%** |
| CXCL1 | C-X-C motif chemokine 1 | **>70%** | **>70%** |
| CXCL10 | C-X-C motif chemokine 10 | **>70%** | **>70%** |
| CXCL11 | C-X-C motif chemokine 11 | **>70%** | **>70%** |
| CXCL5 | C-X-C motif chemokine 5 | **>70%** | **>70%** |
| CXCL6 | C-X-C motif chemokine 6 | **>70%** | **>70%** |
| CXCL9 | C-X-C motif chemokine 9 | **>70%** | **>70%** |
| CST5 | Cystatin D | **>70%** | **>70%** |
| DNER | Delta and Notch-like epidermal growth factor related receptor | **>70%** | **>70%** |
| CCL11 | Eotaxin-1 | **>70%** | **>70%** |
| 4E-BP1 | Eukaryotic translation initiation factor 4E-binding protein 1 | **>70%** | **>70%** |
| FGF-19 | Fibroblast growth factor 19 | **>70%** | **>70%** |
| FGF-21 | Fibroblast growth factor 21 | **<30%** | **>70%** |
| FGF-23 | Fibroblast growth factor 23 | **<30%** | **>70%** |
| FGF-5 | Fibroblast growth factor 5 | **>70%** | **>70%** |
| IL-8 | Interleukin-8 | **>70%** | **>70%** |
| LAP TGF-beta-1 | Latency-associated peptide transforming growth factor β1 | **>70%** | **>70%** |
| LIF | Leukemia inhibitory factor | **<30%** | **<30%** |
| LIF-R | Leukemia inhibitory factor receptor | **>70%** | **>70%** |
| CSF-1 | Macrophage colony-stimulating factor 1 | **>70%** | **>70%** |
| MMP-1 | Matrix metalloproteinase-1 | **>70%** | **>70%** |
| MMP-10 | Matrix metalloproteinase-10 | **>70%** | **>70%** |
| MCP-1 | Monocyte chemotactic protein 1 | **>70%** | **>70%** |
| MCP-2 | Monocyte chemotactic protein 2 | **>70%** | **>70%** |
| MCP-3 | Monocyte chemotactic protein 3 | **<30%** | **<30%** |
| MCP-4 | Monocyte chemotactic protein 4 | **<30%** | **>70%** |
| CD244 | Natural killer cell receptor 2B4 | **30-70%** | **>70%** |
| NT-3 | Neurotrophin-3 | **<30%** | **>70%** |
| NRTN | Neurturin | **<30%** | **<30%** |
| OSM | Oncostatin-M | **<30%** | **>70%** |
| OPG | Osteoprotegerin | **>70%** | **>70%** |
| PD-L1 | Programmed cell death 1 ligand 1 | **>70%** | **>70%** |
| EN-RAGE | Protein S100-A12 | **<30%** | **>70%** |
| Flt3L | Fms-related tyrosine kinase 3 ligand | **>70%** | **>70%** |
| CX3CL1 | Fractalkine | **>70%** | **>70%** |
| GDNF | Glial cell-derived neurotrophic factor | **<30%** | **>70%** |
| HGF | Hepatocyte growth factor | **>70%** | **>70%** |
| IFN-gamma | Interferon gamma | **<30%** | **<30%** |
| IL-1 alpha | Interleukin-1 α | **<30%** | **<30%** |
| IL-10 | Interleukin-10 | **<30%** | **>70%** |
| IL-10RA | Interleukin-10 receptor subunit α | **<30%** | **30-70%** |
| IL-10RB | Interleukin-10 receptor subunit β | **>70%** | **>70%** |
| IL-12B | Interleukin-12 subunit β | **>70%** | **>70%** |
| IL-13 | Interleukin-13 | **<30%** | **<30%** |
| IL-15RA | Interleukin-15 receptor subunit α | **<30%** | **>70%** |
| IL-17A | Interleukin-17A | **<30%** | **30-70%** |
| IL-17C | Interleukin-17C | **<30%** | **30-70%** |
| IL-18 | Interleukin-18 | **>70%** | **>70%** |
| IL-18R1 | Interleukin-18 receptor 1 | **>70%** | **>70%** |
| IL-2 | Interleukin-2 | **<30%** | **<30%** |
| IL-2RB | Interleukin-2 receptor subunit β | **<30%** | **<30%** |
| IL-20 | Interleukin-20 | **<30%** | **<30%** |
| IL-20RA | Interleukin-20 receptor subunit α | **<30%** | **<30%** |
| IL-22 RA1 | Interleukin-22 receptor subunit α-1 | **<30%** | **<30%** |
| IL-24 | Interleukin-24 | **<30%** | **<30%** |
| IL-33 | Interleukin-33 | **<30%** | **<30%** |
| IL-4 | Interleukin-4 | **<30%** | **<30%** |
| IL-5 | Interleukin-5 | **<30%** | **<30%** |
| IL-6 | Interleukin-6 | **30-70%** | **<30%** |
| IL-7 | Interleukin-7 | **>70%** | **>70%** |
| SLAMF1 | Signaling lymphocytic activation molecule | **<30%** | **>70%** |
| SIRT2 | SIR2-like protein 2 | **30-70%** | **>70%** |
| STAMBP | STAM-binding protein | **<30%** | **>70%** |
| SCF | Stem cell factor | **>70%** | **>70%** |
| ST1A1 | Sulfotransferase 1A1 | **<30%** | **<30%** |
| CD6 | T cell surface glycoprotein CD6 isoform | **<30%** | **>70%** |
| CD5 | T-cell surface glycoprotein CD5 | **>70%** | **>70%** |
| TSLP | Thymic stromal lymphopoietin | **<30%** | **<30%** |
| TNFB | TNF-β | **<30%** | **>70%** |
| TRANCE | TNF-related activation-induced cytokine | **<30%** | **>70%** |
| TRAIL | TNF-related apoptosis-inducing ligand | **>70%** | **>70%** |
| TGF-alpha | Transforming growth factor α | **>70%** | **>70%** |
| TWEAK | Tumor necrosis factor (Ligand) superfamily member 12 | **>70%** | **>70%** |
| TNF | Tumor necrosis factor | **<30%** | **<30%** |
| TNFSF14 | Tumor necrosis factor ligand superfamily member 14 | **>70%** | **>70%** |
| TNFRSF9 | Tumor necrosis factor receptor superfamily member 9 | **>70%** | **>70%** |
| uPA | Urokinase-type plasminogen activator | **>70%** | **>70%** |
| VEGF-A | Vascular endothelial growth factor A | **>70%** | **>70%** |

The ninety-two proteins measured are listed above along with their corresponding paired call rate for cerebrospinal fluid (CSF) and plasma. BDNF was excluded due to issues with assay reliability. Proteins with a call rate >70% (green) were used for analysis (# pass: CSF=47, plasma=68).

**Figure S1.** Effects of aerobic exercise on inflammation-related protein levels in cerebrospinal fluid and plasma correcting for variation in sample handling.


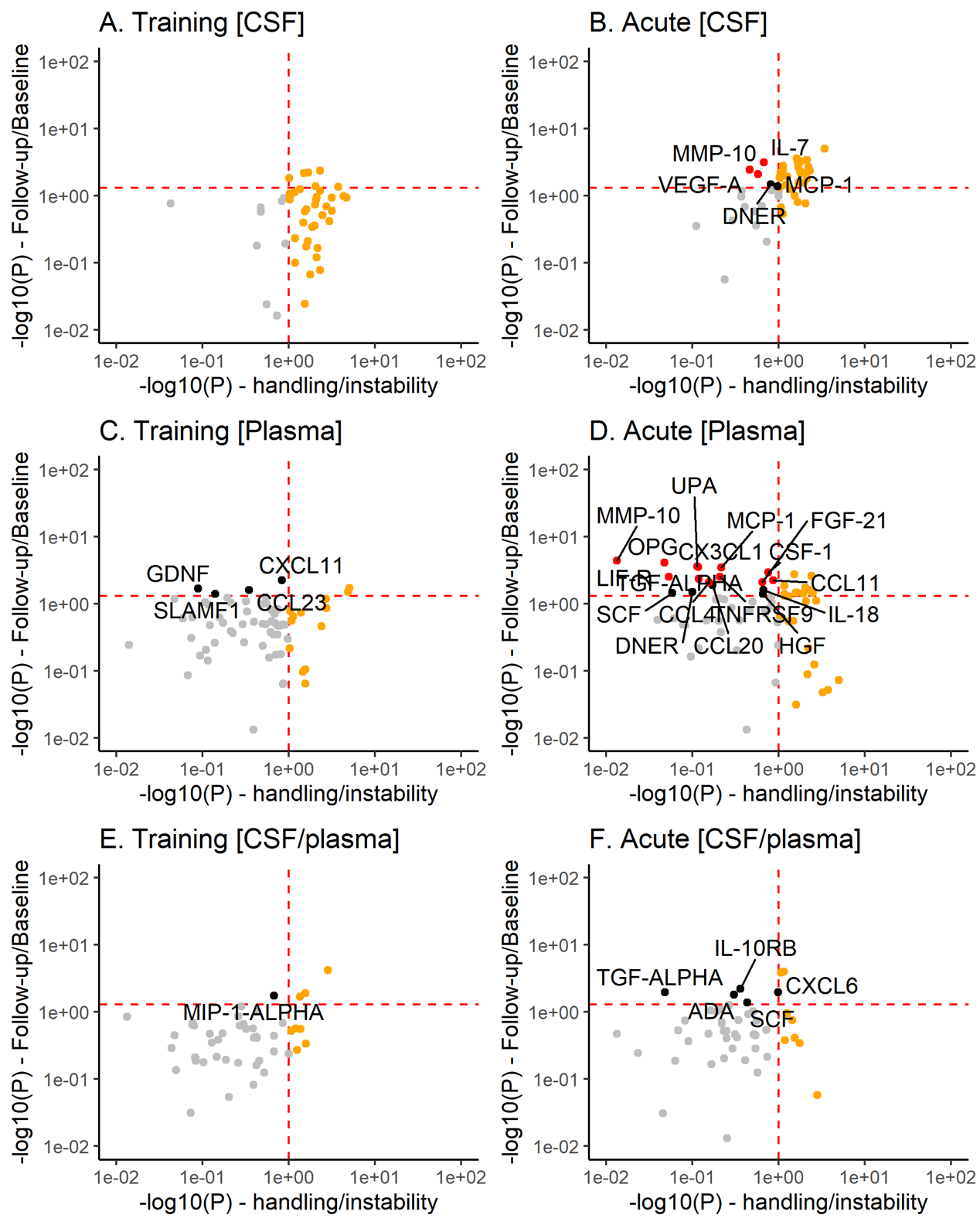


Log base-10 scaled scatterplots illustrating: the association between inflammation- related protein levels and either [A] training or [B] acute exercise in CSF [first row], plasma [2^nd^ row], and the ratio of CSF/plasma [3^rd^ row]; and the association between protein levels and markers of sample handling variability (CSF: CCL19; Plasma: Axin-1). The horizontal red dashed line indicates an exploratory cutoff of P=0.05 and associations with P_FDR_ <0.05 are highlighted red. Measures susceptible to differences in sample handling (P<0.10, red dotted line) are highlighted orange and were either removed (**Fig. 2**) or corrected for handling variability (**Table 3**) to reduce potential bias. R: A language and environment for statistical computing. R Foundation for Statistical Computing, Vienna, Austria. URL [https://www.R-project.org/](https://www.r-project.org/).

**Figure S2.** Correlation between cerebrospinal fluid and plasma metabolites.


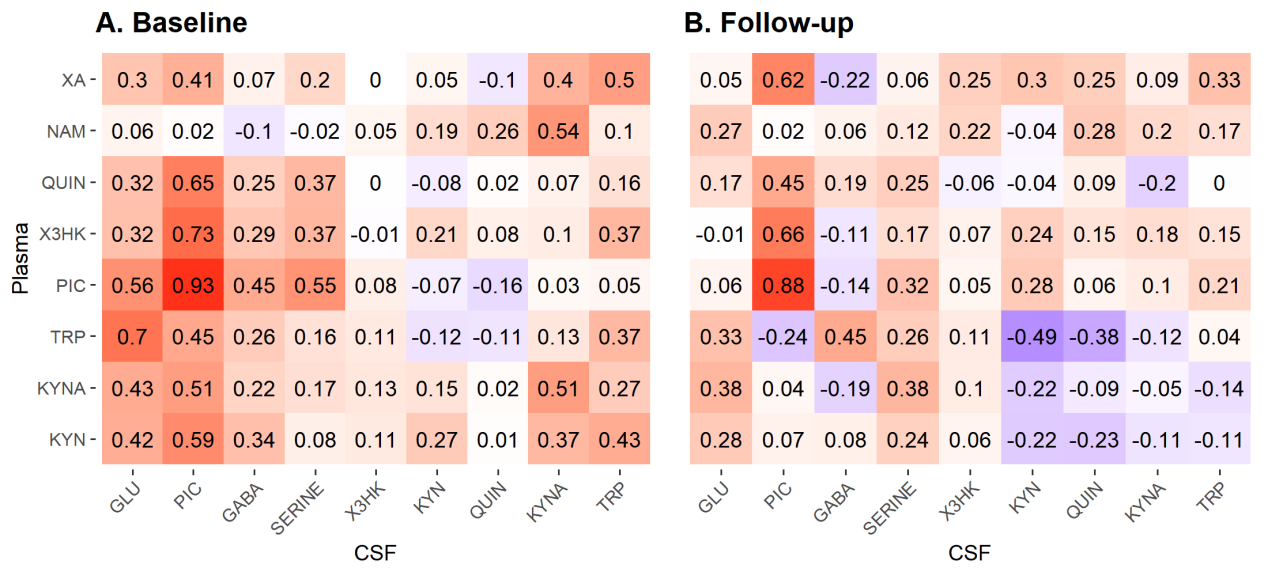


Heatmaps illustrating the correlation between metabolites measured in cerebrospinal fluid (CSF) and those measured in plasma. Pearson correlation coefficients are listed and color-coded for each cell. Analysis was stratified by measures at [A] baseline and [B] follow-up. R: A language and environment for statistical computing. R Foundation for Statistical Computing, Vienna, Austria. URL [https://www.R-project.org/](https://www.r-project.org/).

**Figure S3.** Distribution of glutamate, GABA, and serine in cerebrospinal fluid, stratified by intervention group and sampling time.


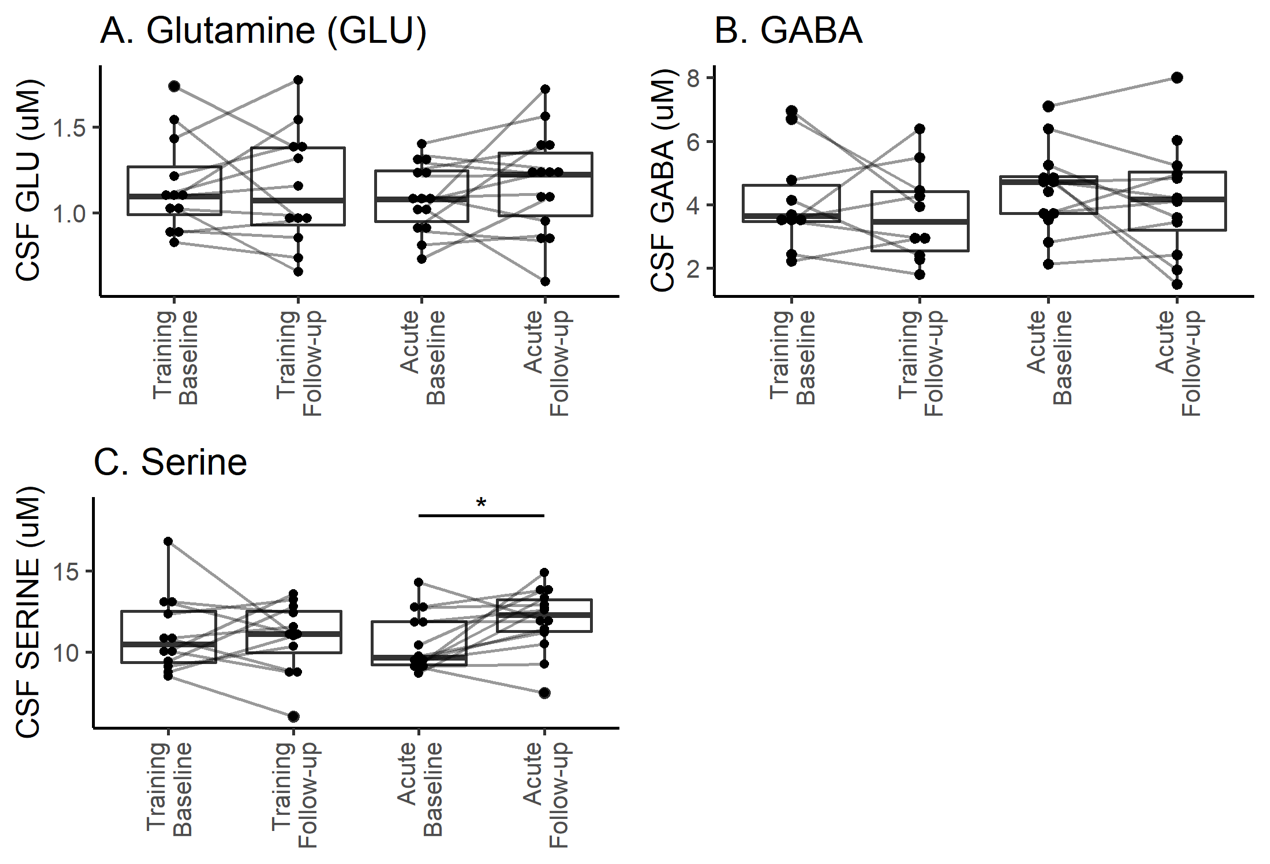


Box and dot plots illustrate metabolite levels (μM) in cerebrospinal fluid (CSF) among paired samples (line) before and after either training or acute aerobic exercise. Significance was determined stratified by intensity (solid bar). Significance levels (P): (*) < 0.05, (**) < 0.01, and (***) < 0.005. R: A language and environment for statistical computing. R Foundation for Statistical Computing, Vienna, Austria. URL [https://www.R-project.org/](https://www.r-project.org/).
